# m6A: a novel strategy for osteoporosis treatment

**DOI:** 10.3389/fcell.2025.1603068

**Published:** 2025-09-08

**Authors:** Chunlu Yan, Xiaolong Xiao, Fan Yang, Yangyang Shi, Qiao Wan, Yan Zhang, Fangyu An

**Affiliations:** ^1^ Dunhuang Medical Academy, Gansu University of Chinese Medicine, Lanzhou, Gansu, China; ^2^ School of Tradional Chinese and Werstern Medicine, Gansu University of Chinese Medicine, Lanzhou, Gansu, China; ^3^ School of Basic Medicine, Gansu University of Chinese Medicine, Lanzhou, Gansu, China; ^4^ School of Health and Health, Lanzhou Modern Vocational College, Lanzhou, Gansu, China; ^5^ Teaching Experiment Training Center, Gansu University of Chinese Medicine, Lanzhou, Gansu, China

**Keywords:** osteoporosis, M6A, mesenchymal stem cell, osteoblast, osteoclast

## Abstract

Osteoporosis (OP) is a systemic metabolic disease characterised by increased bone fragility, with bone loss being the primary cause of its onset and progression. Regulating the dynamic balance between osteoblast (OB) formation and osteoclast-mediated bone resorption is crucial for preventing bone loss in OP. N6-methyladenosine (m6A), the most abundant and common RNA modification, is regulated by various proteins, including m6A methyltransferases, demethylases, and binding proteins. m6A methylation plays a key role in bone metabolism in OP, influencing the osteogenic and adipogenic differentiation of mesenchymal stem cells (MSCs), the osteogenic differentiation and bone formation capacity of OBs, as well as osteoclastic differentiation and resorptive activity. However, the specific molecular mechanisms through which m6A methylation regulates bone metabolism in OP remain incompletely understood. In this review, we comprehensively discuss the structure and function of m6A and summarise the roles of m6A methyltransferases, demethylases, and binding proteins. We also examine the regulatory mechanisms of m6A in MSCs, OBs, and osteoclasts, and discuss associated targeted therapies. This overview of the research on m6A is expected to highlight valuable insights and the translational potential for developing treatment strategies for OP.

## 1 Introduction

Osteoporosis (OP) is a systemic metabolic bone disease characterised by increased bone loss, decreased bone strength, and a heightened risk of fractures (Zhou et al., 2023). This condition exacerbates rates of disability and mortality in older patients (Borgström et al., 2020). Epidemiological data show that more than 200 million people worldwide suffer from OP (De Martinis et al., 2021). It is estimated that by 2050, the number of OP patients aged 50 and over will exceed 400 million globally, with osteoporotic fractures in men and women increasing by 310% and 240%, respectively (Liu et al., 2022). This indicates that the number of patients with OP-related fractures is increasing rapidly, which has imposed a serious economic burden on individuals and society, making it one of the most pressing public health issues. Consequently, the prevention and treatment of OP have become urgent problems in the medical field.

N6-methyladenosine (m6A) was first discovered in 1974 and refers to the methylation modification of adenosine at the sixth position of its nitrogenous base (Desrosiers et al., 1974). Among more than 160 identified RNA post-transcriptional modifications, m6A is the most abundant and common (Boccaletto et al., 2018). Studies have shown that m6A accounts for more than 50% of the total methylated ribonucleotides in mammals and is the most significant messenger RNA (mRNA) modification in these organisms (Wei et al., 1975). m6A regulates RNA metabolism by participating in various processes such as RNA folding and structure, maturation, nuclear export, translation, and decay (He et al., 2019), thereby maintaining normal cellular activities.

Increasing evidence indicates that m6A plays a crucial role in bone metabolism by regulating the osteogenic and adipogenic differentiation of mesenchymal stem cells (MSCs), osteoblast (OB) differentiation, and osteoclast differentiation. It also plays a clear regulatory role in OP; however, the specific regulatory mechanisms underlying m6A activity in OP remain unclear. Recent clinical studies have found (Wang et al., 2024; Tian et al., 2025) that the m6A modification level and METTL3 mRNA and protein expression levels in the trabecular tissue of patients with OP are significantly reduced. Regulating the m6A modification and expression levels of regulatory proteins in patients with OP may be an effective method for the treatment and prevention of OP. Therefore, this review summarises the structural characteristics and functions of m6A, along with the regulatory proteins involved in m6A methylation, including m6A methyltransferases, demethylases, and binding proteins. This review also summarises the regulatory mechanisms underlying m6A methylation modifications in the osteogenic and adipogenic differentiation of MSCs, as well as the regulatory mechanisms underlying OB and osteoclast differentiation. Additionally, the review briefly summarises current therapies targeting m6A methylation to regulate bone metabolism, providing a theoretical basis for the clinical exploration of methods for treating and preventing OP.

## 2 Structural characteristics and functions of m6A and its regulatory proteins

### 2.1 Structure and function of m6A

**FIGURE 1:**
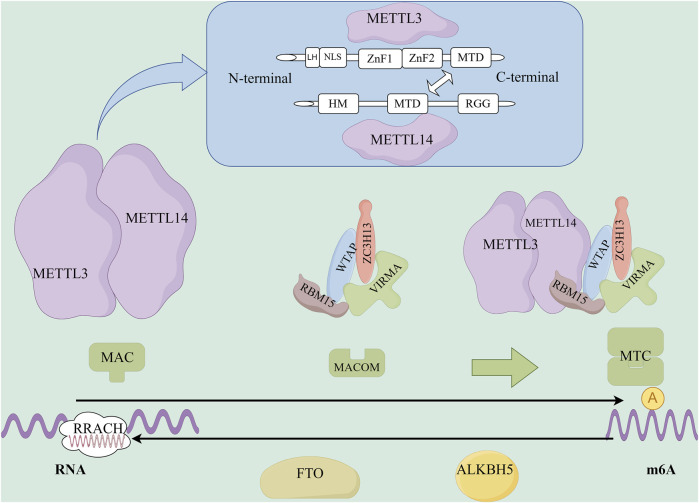
The structure and function of m6A methyltransferase and m6A demethylase. METTL3 is composed of three primary structural domains: the zinc finger domain (ZFD), the methyltransferase domain (MTD), and the N-terminal domain (NTD). METTL14 is constituted by an N-terminal alpha helix motif (HM), the methyltransferase domain (MTD), and a C-terminal arginine-glycine-repeat (RGG) region. Among them, METTL3 has catalytic activity, and METTL14 has the ability to recognize and bind substrate RNA. METTL3/METTL14 form a heterodimer (MAC), which is the catalytic subunit of the m6A methyltransferase. MAC promotes m6A modification of RNA throughout the entire transcription process by recognizing the “RRACH” motif. Additionally, RBM15/ZC3H13/WTAP/VIRMA form the regulatory subunits of the m6A methyltransferase complex (MACOM), which assists in the function of MAC. MAC and MACOM form the m6A methyltransferase complex (MTC), which plays a role in promoting RNA m6A modification throughout the transcription process. The m6A demethylases, including the fat mass and obesity-associated protein (FTO) and ALKB homolog 5 (ALKBH5), are capable of removing the m6A methylation from RNA.

m6A methylation refers to the methylation at the sixth position of the nitrogenous base in adenosine. m6A is widely present in mRNAs or non-coding RNAs (ncRNAs) in viruses, yeast, Arabidopsis, *Drosophila*, mammals, and other organisms. It is the most common RNA modification (Sun et al., 2023). The localisation of genes in the m6A transcriptome can be achieved through m6A-specific methylated RNA immunoprecipitation and high-throughput sequencing (Dominissini et al., 2012; Meyer et al., 2012; Dominissini et al., 2013). Previous sequencing analyses have revealed that m6A modifications are mainly concentrated in the common motif RRACH (DRACH) (Figure 1), where (R = G/A, H = A/C/U) (Wei and He, 2019). m6A modifications are significantly enriched in the 3′ untranslated region (3′ UTR) of the RRACH (DRACH) motif, around the stop codon, and at the long internal exons (Dominissini et al., 2012; Meyer et al., 2012; Ke et al., 2015). Functionally, m6A plays a significant role in RNA metabolism, including pre-mRNA splicing, 3′ end processing, nuclear export, translational regulation, mRNA decay, and ncRNA processing (Sun et al., 2023). In mammals, m6A methylation also plays key roles in embryonic development, neurogenesis, circadian regulation, stress responses, and tumorigenesis (Pan et al., 2018; Niu et al., 2013). These functions highlight the importance of m6A in the regulation of biological activities.

### 2.2 Structural characteristics and functions of m6A regulatory proteins

m6A methylation is regulated by various proteins, including m6A methyltransferases, m6A demethylases, and m6A methylated reading proteins (Jia et al., 2013).

#### 2.2.1 m6A methyltransferase

m6A methyltransferases include methyltransferase-like 3 (METTL3), methyltransferase-like 14 (METTL14), Wilms’ tumour 1-associated protein (WTAP), zinc finger CCCH-type containing 13 (ZC3H13), RNA-binding motif protein 15 (RBM15), and Vir-like m6A methyltransferase-associated protein (VIRMA), among others (Jiang W. et al., 2021). The binding of these m6A methyltransferases can form a stable complex, namely, methyltransferase complex for N6-methyladenosine m6A (MTC), which primarily catalyses the methylation of the N6 position of adenine (A) in RNA molecules, resulting in the formation of m6A. This is one of the most abundant internal chemical modifications found in eukaryotic mRNA. The MTC is primarily composed of a catalytic subunit, the m6A-METTL complex (MAC), and a regulatory subunit, the m6A-METTL-related complex (MACOM), both of which play crucial roles in regulating methyltransferase activity (Liu et al., 2015; Peng et al., 2023). These two complexes interact to regulate m6A deposition in RNA (Figure 1) (Knuckles et al., 2018).

##### 2.2.1.1 m6A-METTL complex (MAC)

The MAC is primarily composed of METTL3 and METTL14. METTL3 consists of three main domains: the zinc finger domain (ZFD), the methyltransferase domain (MTD), and the N-terminal domain (NTD) (Figure 1). The ZFD contains two CCCH-type zinc fingers, ZnF1 and ZnF2, which are connected by antiparallel β sheets (Śledź and Jinek, 2016). These zinc fingers are responsible for recognising and binding RNA molecules (Huang et al., 2019). The MTD adopts a classical α-β-α sandwich fold structure, including a mixed eight-stranded β sheet, four α helices, and three 310 helices on both sides (Wang et al., 2016). This domain can bind to the MTD of METTL14 and is responsible for the specific recognition of m6A modification sites in RNA (Śledź and Jinek, 2016). The NTD consists of a leader helix and nuclear localisation signal region (Śledź and Jinek, 2016) and methylates the secondary structures of different RNAs based on their folding patterns and stability, influencing the activity of methyltransferases and the overall yield of RNA methylation (Meiser et al., 2020). Additionally, the NTD works synergistically with the RGG domain of METTL14 to improve methyltransferase efficiency (Meiser et al., 2020; Yoshida et al., 2022).

METTL14 is composed of three main domains: the N-terminal α-helical motif (HM), MTD, and C-terminal arginine-glycine repeat (RGG) (Figure 1). The RGG repeat at the C-terminal of METTL14 binds to RNA with a specific secondary structure, aiding in RNA substrate recognition (Leder et al., 2020; Zhou et al., 2021). It is the structure necessary for maintaining METTL14 activity.

METTL3 and METTL14 frequently exist in the form of the MAC. The MTDs of both METTL3 and METTL14 form stable heterodimers by binding to loop junctions near the active site of METTL3, facilitating substrate RNA binding (Wen et al., 2018). Crystallographic analyses of MAC have revealed that it binds at a 1:1 ratio to the MTD domains of METTL3 and METTL14 (Lence et al., 2019). Additionally, the MAC can recognise the “RRACH” motif and promote the m6A modification of RNA during transcription (Linder et al., 2015) (Figure 1).

##### 2.2.1.2 m6A-METTL-related complex (MACOM)

The MACOM primarily consists of WTAP, ZC3H13, RBM15, and KIAA1429. WTAP, an auxiliary member of the methyltransferase complex, mainly exists in two complexes: METTL3-METTL14-WTAP and ZC3H13-WTAP-HAKAI. It is the human ortholog of MUM2. Ping et al. (2014) found that the level of m6A in mRNA was significantly reduced after WTAP or METTL3 had been knocked down in cells, indicating that WTAP promotes the activity of m6A methyltransferase. Additionally, the accumulation of METTL3 and METTL14 in nuclear speckles has been found to decrease after the depletion of WTAP, whereas the knockdown of METTL3 or METTL14 had no effect on WTAP. This suggests that WTAP affects m6A levels by promoting the accumulation of METTL3 and METTL14 in nuclear speckles (Meyer and Jaffrey, 2017; Wen et al., 2018). Furthermore, WTAP functions as a bridging molecule between the METTL3-METTL14-WTAP and ZC3H13-WTAP- HAKAI complexes, facilitating m6A methylation by recruiting methyltransferases to the nucleus (Wen et al., 2018).

ZC3H13 is essential for the nuclear localisation of WTAP, HAKAI, METTL3, and METTL14. Knuckles et al. (2018) measured m6A levels in mouse stem cells (mESCs) following the knockout of ZC3H13 and found that m6A enrichment had been significantly reduced. In addition, ZC3H13 blocks m6A methylation by altering the nuclear localisation of WTAP and HAKAI (Wen et al., 2018). These findings suggest that ZC3H13 can regulate the level of m6A methylation.

RBM15 is an adaptor protein in the methyltransferase complex. It can also bind to METTL3 and WTAP, guiding these proteins to specific RNA sites for m6A modification (Wang D. et al., 2020). KIAA1429, another adaptor protein in the VIRMA subfamily, serves as a bridge between METTL3, METTL14, WTAP, ZC3H13, RBM15, and other complex members. KIAA1429 recruits and directs core catalytic components, such as METTL3, METTL14, and WTAP, to specific RNA regions for m6A methylation (Yue et al., 2018).

**FIGURE 2:**
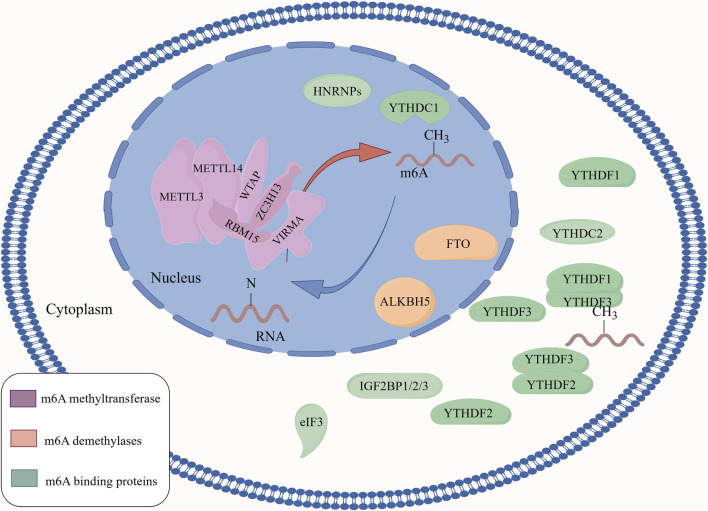
The regulatory mechanism of m6A methylation modification. m6A methyltransferase uses a complex composed of METTL3 and METTL14 as the catalytic core to promote the level of m6A methylation on RNA by interacting with a complex composed of regulatory proteins such as WTAP, ZC3H13, RBM15, and KIAA1429. The m6A demethylases FTO and ALKBH5 regulate the level of m6A on RNA by removing m6A from RNA. m6A-binding proteins are a class of proteins that can specifically bind to m6A modifications, and play an important role in regulating mRNA translation and degradation.

m6A methyltransferases use the MAC as the catalytic core and interact with MACOM (Figure 1). These interactions further promote the activity of m6A methyltransferase *in vivo* and increase the accumulation of m6A methyltransferase in nuclear speckles. The methyltransferase complex can also promote m6A methylation levels by altering the nuclear localisation of WTAP and HAKAI or by recruiting methyltransferase to pre-mRNA. Furthermore, METTL3, METTL14, and WTAP can be recruited and guided to specific RNA regions for m6A methylation, promoting the nuclear deposition of m6A (Figure 2).

#### 2.2.2 m6A demethylase

The primary m6A demethylases are Fat mass and obesity-associated protein (FTO) and AlkB homologous protein 5 (ALKBH5) (Figure 1). FTO is localised in both the nucleus and cytoplasm and belongs to the AlkB family of non-heme Fe(II)/α-ketoglutarate-dependent dioxygenases. FTO consists of two domains: an NTD and a carboxy-terminal domain (CTD). The NTD is the core region responsible for the demethylation activity of FTO and interacts with the CTD to maintain protein stability (Huang et al., 2023). FTO was the first-identified demethylase that promotes the reduction of m6A to adenosine (Jia et al., 2011).

ALKBH5 is mainly localised in the nucleus. ALKBH5 shares a high level of homology with FTO, and both FTO and ALKBH5 catalyse m6A demethylation in a Fe(II)- and α-ketoglutarate-dependent manner through the following steps: 1) m6A demethylase oxidises m6A to form N6-hydroxymethyladenosine (hm6A); 2) hm6A is converted to N6-formyladenosine (f6A); 3) f6A is further converted to adenosine (A), completing the demethylation process (Huang et al., 2023; Wang T. et al., 2020). FTO and ALKBH5 catalyse the m6A demethylation reaction differently. FTO can demethylate m6A on mRNA and U6 RNA, but ALKBH5 only targets m6A methylation on ssRNA (Zheng et al., 2013). Thus, m6A demethylases regulate the level of m6A on RNA by removing the m6A modifications, which in turn regulates the transcription, translation, and degradation of RNA in the cell (Figure 2).

#### 2.2.3 m6A-binding proteins

m6A-binding proteins can be categorised as those containing or lacking YTH domains. m6A-binding proteins containing YTH domains include YTH domain family protein 1 (YTHDF1), YTH domain family protein 2 (YTHDF2), YTH domain family protein 3 (YTHDF3), YTH domain protein 2 (YTHDC2) in the cytoplasm and YTH domain protein 1 (YTHDC1) in the nucleus. The YTH domain is characterised by two tryptophan residues and one leucine residue, which together form a hydrophobic pocket that stably interacts with m6A and specifically recognises and binds to m6A-containing RNA (Secco et al., 2024). m6A-binding proteins lacking YTH domains include three main types: heterogeneous nuclear ribonucleoproteins (HNRNPs), insulin-like growth factor 2 binding proteins (IGF2BP1/2/3), and eukaryotic initiation factor 3 (eIF3) (Song et al., 2022).

The YTHDF family of proteins is mainly localised in the cytoplasm. Upon recognising the m6A site, YTHDF1 also recruits the translation initiation factor eIF3a complex to initiate translation and promote polysome formation, which improves the translation efficiency of target mRNAs and regulates their stability (Chen et al., 2021). Additionally, YTHDF1 interacts with AGO2 *via* its YTH domain to promote P-body formation, thereby accelerating mRNA degradation (Li Y. et al., 2022). YTHDF2 can promote mRNA degradation by reducing mRNA stability (Zhao L. et al., 2024). The results of zebrafish studies by Zhao et al. (2017) confirmed this view and highlighted the recruitment of the CCR4-NOT deadenylase complex by YTHDF2 (Hsu et al., 2017). YTHDF3 cooperates with YTHDF1 to promote the translation of m6A-modified mRNA, likely through interactions with ribosomal 40S/60S subunits (Li et al., 2017). YTHDF3 can also influence the YTHDF2-mediated decay of m6A-modified mRNA (Shi et al., 2017). Upon recognising the m6A site, YTHDF accelerates RNA deadenylation and cleaves RNA by recruiting the CCR4-NOT deadenylase or HRSP12 RNaseP/MRP complexes (Qi et al., 2022; Xu et al., 2022). YTHDC1, located in the nucleus, regulates mRNA splicing by recruiting and regulating pre-mRNA splicing factors to bind to specific regions of targeted mRNAs (Xiao et al., 2016). YTHDC2, located in the cytoplasm, is mainly involved in reducing mRNA abundance and improving target translation efficiency (Hsu et al., 2017).

The HNRNP family of proteins is mainly localised in the nucleus. HNRNPs that can recognise m6A include HNRNP A2B1 (HNRNPA2B1), HNRNP C (HNRNPC), and HNRNP G (HNRNPG). HNRNPA2B1 directly binds and regulates the processing of m6A-modified transcripts by interacting with the DGCR8 protein (Jiang X. et al., 2021). HNRNPC is involved in pre-mRNA processing (Wang L. C. et al., 2020). HNRNPG selectively binds to RNA through the RNA recognition motif (RRM) and RGG motif (Zhou et al., 2019). The HNRNP family of proteins plays a role in gene transcription, splicing, transcriptional stability, mRNA localisation, translation, and gene regulation mediated by microRNAs (miRNAs) and circular RNAs. HNRNP proteins are important for protein interactions, transcriptional activation, and nuclear localisation (Nishanth and Jha, 2024). IGF2BP1/2/3 recognise m6A and promote mRNA stability and translation in an m6A-dependent manner (Wang T. et al., 2020). EIF3, a eukaryotic initiation factor in mammalian cells, mainly recognises and binds m6A modifications in the 5′ UTR of cellular mRNA, enhances ribosome loading and promotes the translation of target gene mRNA, playing an important role in eukaryotic translation (Song et al., 2022).

In summary, m6A-binding proteins comprise a class of proteins that specifically bind to m6A modifications. These m6A regulatory proteins collectively act on RNA by affecting the levels of m6A, thereby influencing various metabolic processes including the recognition of m6A, translation of m6A-modified mRNA, degradation of m6A-modified mRNA, and changes in mRNA stability. Ultimately, they affect the transcription and translation of related genes, regulating the life activities of organisms (Wen et al., 2024) (Figure 2). Table 1 summarises the role of m6A regulatory proteins in m6A methylation.

**TABLE 1:** The functions of m6A regulatory proteins.

Regulatory protein	Adjustment factor	Function	References
m6A methyltransferase	METTL3	Catalytic activity	Huang et al. (2019)
	METTL14	Identify and bind substrate RNA, enhance METTL3 catalytic activity	Knuckles et al. (2018), Huang et al. (2019)
	WTAP	Promote methyltransferase activity, nuclear speckle localization, and nuclear speckle accumulation	Meyer and Jaffrey (2017), Wen et al. (2018)
	ZC3H13	Alter the nuclear localization of WTAP, HAKAI, METTL3, and METTL14	Wen et al. (2018)
	RBM15	Adapter proteins, recruiting methyltransferases to precursor mRNA.	Wang D. et al. (2020)
	KIAA1492	Adapter proteins recruit and guide catalytic core components (such as METTL3/METTL14/WTAP) to specific RNA regions for m6A methylation	Yue et al. (2018)
m6A demethylases	FTO	Demethylate m6A on mRNA and U6 RNA.	Zheng et al. (2013)
	ALKBH5	Demethylation of m6A on ssRNA.	Zheng et al. (2013)
m6A binding proteins	YTHDF1	The recruitment of the translation initiation factor eIF3a complex promotes translation initiation, facilitates the formation of polysomes, enhances the translation efficiency of target mRNAs, and regulates the stability of target mRNAs	Chen et al. (2021), Li Y. et al. (2022)
	YTHDF2	Reduce mRNA stability, promote mRNA degradation	Zhao L. et al. (2024)
	YTHDF3	Synergizes with YTHDF1 to promote the translation of m6A-modified mRNA; affects the decay of mRNA mediated by YTHDF2 through m6A methylation modification	Li et al., 2017 Shi et al. (2017)
	YTHDC1	Recruitment and regulation of precursor mRNA splicing factors regulate mRNA splicing	Xiao et al. (2016)
	YTHDC2	Reduce target abundance, enhance target translation efficiency	Hsu et al. (2017)
	HNRNP	HNRNPA2B1 binds and regulates the processing of m6A-modified transcripts; HNRNPC processes precursor mRNAs; HNRNPG selectively binds RNA.	Jiang W. et al. (2021), Wang L. C. et al. (2020), Zhou et al. (2019)
	IGF2BP1/2/3	Identify m6A and promote mRNA stability and translation in an m6A-dependent manner	Wang T. et al. (2020)
	eIF3	Recognition and binding of m6A modifications in the 5′UTR of cellular mRNA enhance ribosomal loading and promote the translation of target gene mRNA.	Song et al. (2022)

The function of m6A regulatory proteins is strictly dependent on the presence of m6A modifications on RNA. m6A methyltransferases, demethylases, and binding proteins regulate the m6A modification levels of mRNA through a dynamic cycle of increasing, decreasing, and recognising these modifications. This precise regulation of the mRNA life cycle impacts cellular gene expression and function.

## 3 Regulatory mechanisms of m6A in OP

Bone growth and maintenance primarily involve OBs (bone-forming cells) and osteoclasts (bone-resorbing cells), and their activities are strictly controlled by environmental factors, hormones, and epigenetic and mechanical stresses (Ponzetti and Rucci, 2021). During biological growth, OBs continuously undergo a deposition reaction, while osteoclasts undergo a resorption reaction. Through this ongoing process of deposition and absorption, the bone undergoes mechanical “remodelling,” during which OB formation exceeds osteoclast resorption, and bone mass accumulates to meet the needs of body growth and development (Peng et al., 2020). At biological maturity, bone mass accumulation reaches its peak. During this phase, the formation of OBs is largely balanced by resorption from osteoclasts, thus maintaining the dynamic balance of bone mass (Karsenty and Khosla, 2022; Florencio-Silva et al., 2015). However, with increasing age, abnormal hormone secretion, and other factors, OB-dominated bone formation weakens, and osteoclast-dominated bone resorption increases, eventually leading to osteopenia or OP (Li and Tian, 2025; Weivoda and Bradley, 2023; Florencio-Silva et al., 2015). Bone loss is the primary cause of OP onset and progression, and regulating the dynamic balance between OB formation and resorption is key to preventing OP.

In recent years, significant progress has been made in understanding the relationship between m6A and OP (Wu et al., 2018; Yu et al., 2020). METTL3, one of the methyltransferases of m6A, has been found to promote the osteogenic differentiation of bone marrow MSCs and inhibit adipogenic differentiation (Wu et al., 2018), while in MC3T3-E1 cells, it has been shown to inhibit osteogenic differentiation (Yu et al., 2020; Mi et al., 2020). Table 2 summarises the roles of m6A family proteins in different cells.

**TABLE 2:** Regulatory mechanisms of m6A in osteogenic, adipogenic, and osteoclastic differentiation in different cell.

	Cell	Adjustment factor	Signal pathway/target	Marker molecules of osteogenic, adipogenic and osteoclastic	Function	References
Mesenchymal stem cells	BMSC	METTL3↑	VEGF↑	VEGFA-164↑, VEGFA-188↑	Osteogenic (+)	Carmeliet et al. (1999), Jiang X. et al. (2021)
	BMSC	METTL3↑	m6A-pre-miR-320↑/miR-320↓-RUNX2↑	pre-miR-320/miR-320↓, RUNX2↑	Osteogenic (+)	Yan et al. (2020)
	MenSC	METTL3↑	MYD88-RNA↑/MYD88↑/NF-κB↑	RUNX2↓, SP7↓, ALP↓, m6A-MYD88-RNA↑, NF-κB↑	Inhibit osteogenesis (−)	Yu et al. (2020)
	BMSC	METTL14↑	IGF2BP/beclin-1↑	BGLAP2↑, BMP2↑, RUNX2↑,SPP1↑, beclin-1↑	Osteogenic (+)	He et al. (2022)
	BMSC	FTO↑	RUNX2↓	RUNX2↓, SP7↓, ALP↓, BGLAP↓	Inhibit osteogenesis (−)	Wang W/ et al. (2021)
	C3H10T1/2	FTO↑	AMPK↑	Dlx5↑, RUNX2↑, ALP↑	Osteogenic (+)	Son et al. (2020)
	MSC	ALKBH5↑	PRMT6↓-PI3K/AKT↓	GALAP↓, ALP↓, RUNX2↓, SP7↓	Inhibit osteogenesis (−)	Li et al. (2021)
	hMSC	ALKBH5↑	COL1A1↑,OSX↑,RUNX2↑	COL1A1↑, OSX↑, RUNX2↑	Osteogenic (+)	Weng et al. (2023)
	BMSC	YTHDF1↑	VDAC3↑	VDAC3↑, RUNX2↑, COL1A1↑, ALP↑	Osteogenic (+)	Huang et al. (2024)
	BMSC	METTL3↑	PTH-PTH1R↑	PTH1R↑, PTH↑, MAT↓	Osteogenic (+), Inhibit fat synthesis (−)	Yan et al. (2020), Wu et al. (2018)
	BMSC	WTAP↑	YTHDC1↑/miR-181a and miR-181c↑/SFRP1↓	COL1↑, RUNX2↑, ALP↑, BMP2↑, OPN↑, LPL↓, AP2↓, C/EBP-α↓, C/EBP-β↓, PPAR-γ↓	Osteogenic (+), Inhibit fat synthesis (−)	You et al. (2023)
	BMSC	FTO↑	miR-149-3p↓	ALP↑, BGLAP↑, SPP1↑, COL1A1↑, BMP4↑, CEBPA↓, CEBPB↓, CEBPD↓, FABP4↓, PPARG↓	Osteogenic (+), Inhibit fat synthesis (−)	Li J. et al. (2019)
	MSC	ALKBH5↑	TRAF4↑	ALP↑, RUNX2↑, OCN↑PPAR-γ↓, C/EBP-α↓, FABP4↓	Osteogenic (+), Inhibit fat synthesis (−)	Li Y. et al. (2019), Cen et al. (2020)
Osteoblast	MC3T3-E1	METTL3↑	miR-7212-5p↑/FGFR3↓	BV↓, TV↓, BMD↓, BMP2↓, RUNX2↓	Inhibit osteogenesis (−)	Mi et al. (2020)
	Osteoblast	FTO↑	HSPA1A↑/NF-κB↓	Apoptosis of osteoblasts↓	Osteogenic (+)	Zhang et al. (2019)
	Osteoblast	ALKBH5↑	RUNX2↑	ALP↑, ALP↑, BSP↑, COL1A1↑, RUNX2↑, SP7↑	Osteogenic (+)	Feng et al. (2021)
Osteoclast	Osteoclast	METTL3↑	circ_0008542↑	CTSK↑, MMP9↑, TRAP↑	Promotes osteoclast differentiation (+)	Wang J. et al. (2021)
	Osteoclast	ALKBH5↑	circ_0008542↓	Osteoclast differentiation and bone resorption↓	Inhibits osteoclast differentiation (−)	Wang J. et al. (2021)

Note: ↑, upregualation; ↓, downregualation; (+), promotion; (−), inhibition.

### 3.1 Regulatory mechanisms of m6A in MSCs

MSCs are a class of adult stem cells with multi-directional differentiation potential and can differentiate into a variety of mesodermal-derived cells, whose differentiation potential largely depends on specific factors present in the microenvironment. They can differentiate into OBs, chondrocytes, and bone marrow adipocytes under certain conditions (Capulli et al., 2014). The balance between the osteogenic and adipogenic differentiation of MSCs is extremely important for maintaining bone health (Zhao Y. et al., 2024). With aging or other pathological stimuli, such as hormonal disorders, the adipogenic differentiation of stem cells tends to be greater than osteogenic differentiation, resulting in progressive bone loss (Ouyang et al., 2022; Wang D. et al., 2020). When the adipogenic differentiation of MSCs is less than osteogenic differentiation, MSCs can correct the imbalance in bone metabolism during OP (Xu et al., 2024; Qin et al., 2024). Recent research has indicated that m6A plays a role in regulating osteoporotic bone metabolism by influencing the adipogenic and osteogenic differentiation of MSCs (Cai et al., 2022). Bone marrow mesenchymal stem cells (BMSCs) is a kind of MSCs, which refers to the mesenchymal stem cells derived from bone marrow. They exist in the bone marrow microenvironment for a long time and play an important role in regulating bone metabolism (Wang et al., 2022).

#### 3.1.1 METTL3 promotes bone formation in OBs through m6A methylation of VEGF in BMSCs (contributing to bone formation)

Bone formation occurs *via* two different processes: endochondral ossification and intramembranous ossification. Endochondral bone formation requires the involvement of OBs and is accompanied by the progressive neovascularisation of bone growth (Tong et al., 2019). Osteogenesis and angiogenesis are highly coupled during bone formation, and angiogenesis is a prerequisite for bone tissue regeneration (Fu et al., 2020; Ramasamy et al., 2014); this phenomenon is known as angiogenesis-osteogenesis coupling (Kumar et al., 2022). BMSCs are MSCs derived from bone marrow. They reside in the bone marrow microenvironment for a long time and play an important role in regulating bone metabolism. BMSCs not only differentiate directly into endothelial cells to participate in the formation of new blood vessels in the bone defect area but also secrete angiogenic factors, such as VEGF and angiopoietin, to promote local angiogenesis (Tian et al., 2019). VEGF plays a crucial role in bone formation (Schall et al., 2020; Hu and Olsen, 2016). Carmeliet et al. (1999) specifically knocked down METTL3 in BMSCs and performed osteogenic induction culture. The results showed that the expression level of VEGFA in BMSCs decreased significantly, indicating that METTL3 promotes the osteogenic differentiation of BMSCs by targeting the expression level of VEGF. Three homologous splicing variants of VEGF—VEGFA-120, VEGFA-164, and VEGFA-188—participate in angiogenesis and osteogenesis in BMSCs, but their effects are quite different (Tian et al., 2019). METTL3 knockdown significantly inhibited the mRNA levels of VEGFA-164 and VEGFA-188, which are related to BMSC proliferation and differentiation, but did not significantly alter the mRNA levels of VEGFA-120 (Carmeliet et al., 1999). VEGFA-188 promotes the osteogenic differentiation of BMSCs (Carmeliet et al., 1999). VEGFA-164 can improve BMSC proliferation and cartilage differentiation, and VEGFA-120 has limited involvement in the proliferation and differentiation of BMSCs. These results suggest that METTL3 knockdown inhibits the expression of VEGFA, which facilitates the osteogenic differentiation of BMSCs, and that the low expression of VEGFA in turn inhibits the osteogenic differentiation of BMSCs. Reversing the low expression of METTL3 may be a promising approach to promote the osteogenic differentiation of BMSCs. Additionally, Jiang W. et al. (2021) confirmed that METTL3 regulates bone formation through the m6A methylation of VEGF in BMSCs, thereby corroborating the findings of Carmeliet et al. These findings contribute to the understanding of the epigenetic mechanisms involved in the osteogenic differentiation of BMSCs and provide a promising perspective for innovative therapeutic strategies for chronic bone diseases.

#### 3.1.2 METTL3 promotes the osteogenic differentiation of BMSCs through the m6A-pre-miR-320/miR-320-Runx family transcription factor 2 axis (contributing to bone formation)

Studies have found (Yan et al., 2020) that pre-miR-320 is a target of METTL3 in BMSCs and that METTL3 positively regulates the m6A methylation level of pre-miR-320. Additionally, the expression level of pre-miR-320 positively regulates the expression of miR-320. miR-320 may act as a negative regulator of RUNX2 expression, thereby affecting the osteogenic differentiation of BMSCs. Furthermore, METTL3 can directly promote the m6A methylation of the osteogenesis-related RUNX2 mRNA, enhancing its stability and promoting the expression of RUNX2 in BMSCs, thus promoting the osteogenic differentiation of BMSCs (Yan et al., 2020). METTL3 therefore promotes bone formation in BMSCs by inhibiting the upregulation of RUNX2 levels *via* pre-miR-320 and miR-320 and by maintaining higher levels of RUNX2 expression through the dual mechanism of m6A methylation in RUNX2. The m6A-pre-miR-320/miR-320-RUNX2 axis may therefore be an effective target for OP prevention and treatment.

#### 3.1.3 METTL3 negatively regulates the osteogenic differentiation of MSCs through the MYD88-RNA/MYD88/NF-κB signalling axis (inhibiting osteogenesis)

Yu et al. (2020) isolated MSCs from the menstrual blood of female donors and used osteogenic media for osteogenic induction. The results indicated that METTL3 inhibits the osteogenic differentiation of MSCs by enhancing NF-κB signalling. After the knockdown of METTL3, the expression of MYD88 and the m6A methylation level of MYD88-RNA was significantly inhibited. The activation of NF-κB signalling can inhibit osteogenic differentiation (Swarnkar et al., 2014). MyD88 is a widely accepted NF-κB regulator and has a positive effect on the activation of NF-κB signal transduction (Zhang et al., 2022). Therefore, in MSCs, METTL3 is presumed to positively regulate the expression of MyD88 by controlling the m6A methylation level of MyD88-RNA, which in turn leads to the activation of NF-κB signal transduction and ultimately inhibits bone formation. METTL3, one of the key regulators of osteogenic differentiation in MSCs, may therefore negatively regulate osteogenic differentiation through the METTL3/MyD88-RNA/MyD88/NF-κB signalling axis. Therefore, the targeted regulation of METTL3 expression may be an effective strategy for the prevention and treatment of OP.

#### 3.1.4 METTL14 induces autophagy and osteogenic differentiation of BMSCs through the IGF2BP/Beclin-1 signalling axis (contributing to bone formation)

Previous studies have found that autophagy deficiency can aggravates the bone loss associated with ageing and decreased estrogen levels in women (Shang et al., 2021). The development of OP is usually accompanied by autophagy disorders, which can lead to OB defects in BMSCs (Wang C. et al., 2023). He et al. (2022) reported that the overexpression of METTL14 can positively regulate osteogenic differentiation in OP. The study further revealed that the m6A-binding proteins IGF2BP1/2/3 can recognise m6A-methylated Beclin-1 mRNA in RAW264.7 cells, enhancing the stability of the methylated mRNA and further promoting translation (He et al., 2022). This, in turn, promotes the expression of Beclin-1, an autophagy biomarker, and induces autophagy in BMSCs. Autophagy activates the expression of osteogenesis-related genes such as bone gamma carboxyglutamate protein 2 (BGLAP2), bone morphogenetic protein 2 (BMP2), RUNX2, and secreted phosphoprotein 1 (SPP1), thereby enhancing the formation of BMSCs and promoting bone formation (He et al., 2022). The METTL14/IGF2BP/Beclin-1 signalling axis therefore plays a crucial role in the osteogenic differentiation of BMSCs. In the future, METTL14 may be used as a diagnostic marker and potential therapeutic target for OP.

#### 3.1.5 FTO-mediated RUNX2 methylation negatively regulates osteogenic differentiation of BMSCs (inhibiting osteogenesis)

Wang J. et al. (2021) found that the m6A methylation level of RNA in BMSCs from patients with OP was significantly downregulated, whereas FTO expression was significantly upregulated, suggesting that FTO may participate in the occurrence and development of OP through m6A methylation. This study indicates that FTO inhibits the osteogenic differentiation of BMSCs (Wang J. et al., 2021). The results also showed that the methylation, mRNA, and protein levels of RUNX2 in BMSCs overexpressing wild-type FTO were significantly decreased, and the RNA decay rate was significantly increased. In contrast, the methylation, mRNA, and protein levels of RUNX2 and the RNA decay rate in BMSCs with mutated (R96Q demethylase activity mutation) FTO overexpression were not affected (Wang J. et al., 2021). These results indicate that FTO negatively regulates the methylation level of RUNX2 and promotes RNA decay through its m6A demethylase activity. An OVX mouse model was also established in the study. After the administration of an FTO inhibitor to the OVX mice, the bone mineral density (BMD), bone volume/total volume (BV/TV), and trabecular bone (TB) were significantly increased, while trabecular separation (TB.sp) and the structure model index were significantly decreased, demonstrating that inhibiting FTO expression can reverse bone loss in OP (Wang W. et al., 2021). FTO overexpression therefore inhibits the expression of the osteogenesis-related genes RUNX2, Sp7, ALP, and BGLAP2 in BMSCs through its demethylase activity, thereby inhibiting osteogenic differentiation and promoting the occurrence and development of OP. The downregulation of FTO expression can effectively prevent bone loss in OP, and FTO may be a potential target for the treatment of OP.

#### 3.1.6 FTO mediates the AMPK pathway to positively regulate the osteogenic differentiation of C3H10T1/2 cells (contributing to bone formation)

Son et al. (2020) transfected C3H10T1/2 cells with a plasmid containing FTO (PCMV-FTO) and found that FTO positively regulated osteogenic differentiation of C3H10T1/2 cells. Additionally, treatment with BMP2 significantly increased FTO mRNA expression in a dose- and time-dependent manner, indicating that BMP2 promotes osteogenic differentiation while upregulating FTO expression in C3H10T1/2 cells (Son et al., 2020). C3H10T1/2 cells were transfected with PCDNA3.0-CMyc-MAMPK-A312, a plasmid encoding constitutively active AMPK (Son et al., 2020). The results showed that the mRNA and protein expression of FTO were upregulated, and ALP activity was enhanced. Furthermore, after introducing the AMPK inhibitor compound C (COM.C) into BMP2-treated C3H10T1/2 cells, the expression of FTO, RUNX2, Dlx5, and p-AMPK was downregulated, and the activity of ALP was weakened (Son et al., 2020). These results indicate that AMPK promotes the expression of the osteogenesis-related genes RUNX2 and Dlx5 by upregulating the expression of FTO in C3H10T1/2 cells, thereby promoting osteogenic differentiation. Therefore, AMPK may be a key target in the regulation of FTO expression in C3H10T1/2 cells. Moreover, p-AMPK induces mild endoplasmic reticulum (ER) stress by upregulating FTO expression in C3H10T1/2 cells. BMP2 may therefore induce mild ER stress in C3H10T1/2 cells. These findings suggest a positive relationship between FTO, p-AMPK, and mild ER stress in C3H10T1/2 cells.

In summary, FTO and p-AMPK exhibit bidirectional positive feedback regulation, enhancing osteogenic differentiation of C3H10T1/2 cells. FTO and p-AMPK positively regulate osteogenic differentiation by inducing mild ER stress. FTO may therefore be a key player in the regulation of osteogenic differentiation.

#### 3.1.7 ALKBH5 negatively regulates the osteogenic differentiation of MSCs through the PRMT6-PI3K/AKT axis (inhibiting osteogenesis)

Li et al. (2021) have reported that ALKBH5 negatively regulates the m6A levels and ALP activity of MSCs in the femur tissue of mice. In mice with silenced ALKBH5, the osteogenic differentiation of MSCs was accompanied by a continuous increase in the overall m6A mRNA levels. ALP activity was enhanced as a result of the loss of ALKBH5 in MSCs, and the expression levels of RUNX2 and SP7 in MSCs were increased. These results confirmed that silencing ALKBH5 promoted the osteogenic differentiation of MSCs. After transfecting MSCs with a wild-type ALKBH5 lentivirus to overexpress ALKBH5, the expression of RUNX2 and Sp7 was inhibited, and ALP activity decreased. However, the expression of RUNX2 and Sp7 was not affected by the overexpression of ALKBH5 using a mutated lentivirus lacking ALKBH5 demethylation activity (Li et al., 2021). These results indicated that ALKBH5 inhibited the expression levels of the osteogenesis-related genes RUNX2 and Sp7 through its m6A demethylation activity, thereby negatively regulating the osteogenic differentiation of MSCs. The expression of PRMT6 was positively regulated by the methylation level of PRMT6 but negatively regulated by ALKBH5 (Li et al., 2021), and the negative regulation was mainly achieved by affecting its mRNA degradation rate. PRMT6 regulates the activation of the PI3K/AKT signalling pathway (Jiang et al., 2020; Li et al., 2020). The PI3K/AKT pathway plays an important role in regulating the osteogenic differentiation of stem cells (Huang et al., 2021). Li et al. (2021) further showed that the ALKBH5-PRMT6 axis could control the activation of the AKT signalling pathway and regulate the osteogenic differentiation of MSCs.

PRMT6 therefore positively regulates the osteogenic differentiation of MSCs, while ALKBH5 inhibits the expression of PRMT6 by reducing the methylation level of PRMT6 mRNA and accelerating the degradation rate of PRMT6 mRNA. This, in turn, affects the activation of the PI3K/AKT pathway and negatively regulates the osteogenic differentiation of MSCs. The ALKBH5-PRMT6-PI3K/AKT axis is a key discovery in the regulatory pathway underlying the osteogenic differentiation of MSCs, which has significance in the clinical regulation of bone metabolism in patients with OP.

#### 3.1.8 ALKBH5-mediated m6A demethylation modification promotes osteogenic differentiation of MSCs (contributing to bone formation)

Weng et al. (2023) have found that ALKBH5 expression is significantly downregulated in mice with peri-implantitis combined with type II diabetes. The transfection of human MSCs (hMSCs) with siRNA to downregulate ALKBH5 expression showed that the m6A level of hMSCs increased, while the expression of the osteogenic differentiation-related genes COL1A1, OSX, and RUNX2 significantly decreased. These results indicate that low ALKBH5 expression inhibits the expression of the osteogenic differentiation-related genes COL1A1, OSX, and RUNX2 in hMSCs by promoting m6A methylation in hMSCs, which further inhibits the osteogenic differentiation of hMSCs (Weng et al., 2023). ALKBH5 negatively regulates m6A levels in hMSCs and positively influences osteogenic differentiation. Therefore, it can be speculated that the total m6A level in hMSCs may be negatively correlated with their osteogenic differentiation. In this study (Weng et al., 2023), lentiviruses were used to transfect hMSCs to overexpress ALKBH5. The results showed that the m6A level in hMSCs was significantly downregulated, the mineralisation of the extracellular matrix was enhanced, and the expression of the osteogenic differentiation-related genes COL1A1, OSX, and RUNX2 was significantly upregulated. These findings confirm the negative regulation of m6A levels on osteogenic differentiation in hMSCs. ALKBH5 promoted osteogenic differentiation in hMSCs by inhibiting m6A levels. Therefore, ALKBH5 regulation of osteogenic differentiation of MSCs through m6A demethylation may represent an effective target in the clinical prevention and treatment of OP.

#### 3.1.9 YTHDF1 regulates the osteoblastic differentiation of BMSCs by targeting VDAC3 (contributing to bone formation)

Huang et al. (2024) found that in BMSCs with low VDAC3 expression, the number of senescent cells increased, and the expression of RUNX2 and COL1A1, which are associated with OB differentiation, along with ALP activity and calcium deposition, significantly decreased. The overexpression of VDAC3 in BMSCs resulted in the opposite outcomes, indicating that the overexpression of VDAC3 can inhibit the senescence of BMSCs and promote OB differentiation. Studies have found that the mRNA of VDAC3 is modified by m6A methylation. The overexpression of m6A-binding protein YTHDF1 can enhance the stability of VDAC3 mRNA in BMSCs and significantly increase the protein expression of VDAC3. The luciferase activity of the wild-type VDAC3 construct was significantly increased, whereas the luciferase activity of the mutant construct was not affected, indicating that YTHDF1 is the m6A methylation binding protein of VDAC3 (Huang et al., 2024). These findings suggest that YTHDF1 directly targets and binds to VDAC3. In summary, YTHDF1 can promote BMSC osteogenic differentiation by targeting and regulating VDAC3.

#### 3.1.10 METTL3 promotes osteogenic differentiation and inhibits adipogenic differentiation of BMSCs through the PTH-PTH1R signalling axis (promoting bone formation and inhibiting adipogenesis)

METTL3 was the first m6A methyltransferase to be discovered. The overexpression of METTL3 can significantly reduce the BV/TV, BMD, and TB in OVX OP model mice, elevate TB. sp, and significantly increase bone mass in OVX model mice (Yan et al., 2020). In this study, the CRISPR/Cas9 system was used to establish a METTL3-knockdown mouse model. The data strongly demonstrate that METTL3 has a positive regulatory effect on the osteogenic differentiation of BMSCs (Yan et al., 2020). The overexpression of METTL3 is presumed to be the key mechanism promoting BMSC osteogenic differentiation, while low expression or knockout of METTL3 reverses the promotion of BMSC osteogenic differentiation, which is one of the key factors leading to bone loss. Wu et al. (2018) also found that the loss of METTL3 in MSCs resulted in decreased ALP staining activity, decreased calcium mineralisation deposition, the downregulated expression of osteogenic markers, increased fat deposition indicated by Oil red-O staining, and the upregulated expression of adipogenic factors, indicating that the loss of METTL3 in MSCs leads to impaired bone formation, decreased osteogenic differentiation, and enhanced bone marrow adipogenic differentiation. The results of *in vivo* experiments showed that METTL3 overexpression mainly inhibited the occurrence and development of OP by promoting osteogenic differentiation (Wu et al., 2018). The *in vivo* results therefore proved that METTL3 overexpression is a key target for inducing osteogenic differentiation in OP.

Further studies revealed (Wu et al., 2018) that the mRNA expression of the parathyroid hormone 1 receptor (PTH1R) was hardly affected by METTL3 deletion but that PTH1R protein synthesis was inhibited following METTL3 deficiency, indicating that m6A methylation mainly affects PTH1R expression at the translational level (Fan et al., 2017; Ono et al., 2016). The study also found that in METTL3-knockout MSC cells, the relative distribution of PTH1R mRNA shifted from the polysomal region to the subpolyribosomal region, indicating that the loss of METTL3 led to a decrease in the translational efficiency of PTH1R mRNA (Wu et al., 2018). Other studies found that PTH and PTH1R gene expression was downregulated in METTL3-depleted BMSCs and that the insufficient expression of PTH1R could block the PTH-PTH1R signalling pathway, which in turn dysregulated both the osteogenic and adipogenic differentiation of BMSCs, leading to bone loss (Wu et al., 2018; Tian et al., 2019). These results demonstrate that the expression levels of PTH1R and PTH are strictly regulated by m6A methylation (Wu et al., 2018). METTL3 knockout attenuates PTH-induced osteogenesis. In addition to significantly promoting osteogenic differentiation, PTH inhibits adipocyte differentiation and promotes bone formation. PTH1R is a key regulator of lineage allocation in BMSCs and OB precursors, and PTH1R is also a key receptor for PTH. These results preliminarily confirm that the PTH-PTH1R signalling axis may be a key pathway for METTL3-mediated m6A methylation to regulate the osteogenic and adipogenic differentiation of BMSCs (Wu et al., 2018).

In summary, the overexpression of METTL3 in BMSCs promotes osteogenic differentiation, whereas the deletion of METTL3 inhibits osteogenic differentiation, promotes adipogenic differentiation, and eventually leads to bone loss and OP. The underlying mechanism of action may be related to the PTH-PTH1R signalling axis, which could be the key pathway of METTL3-mediated m6A methylation regulating the osteogenic and adipogenic differentiation of BMSCs.

#### 3.1.11 WTAP promotes osteogenic differentiation and inhibits adipogenic differentiation of BMSCs through the YTHDC1/miR-181a and miR-181c/SFRP1 signalling axes (promoting bone formation and inhibiting adipogenesis)

WTAP is a member of the m6A methyltransferase family. You et al. (2023) found that the expression of WTAP in the bone tissue from patients with OP and from OVX mice was significantly lower than in the normal control groups, while the expression of WTAP increased during the osteogenic induction of BMSCs, suggesting that the expression of WTAP may be related to the osteogenic differentiation of BMSCs. In BMSCs overexpressing WTAP, ALP activity, extracellular matrix mineralisation, and the expression of osteogenesis-related genes including type I collagen (COL1), RUNX2, ALP, BMP2, and osteopontin were significantly increased, indicating that WTAP plays a positive regulatory role in the osteogenic differentiation of BMSCs (You et al., 2023). The mRNA and protein expression levels of adipogenesis-related genes, including lipoprotein lipase, adipocyte-specific fatty acid binding protein, CCAAT/enhancer binding protein alpha (C/EBP-α), CCAAT/enhancer binding protein beta, and peroxisome proliferator-activated receptor gamma (PPAR-γ) were further decreased in BMSCs, indicating that WTAP plays a negative regulatory role in the adipogenic differentiation of BMSCs (You et al., 2023). These results demonstrate that WTAP is an effective molecule to promote osteogenic differentiation and inhibit adipogenic differentiation in BMSCs.

Further osteogenic induction was performed in BMSCs overexpressing WTAP (You et al., 2023). The results showed that WTAP promoted BMSC osteogenic differentiation and inhibited BMSC adipogenic differentiation by positively regulating the expression of miR-181a and miR-181c. YTHDC1 recognised and bound pri-miR-181a and pri-miR-181c after m6A methylation in BMSCs (You et al., 2023). YTHDC1 promotes the expression of methylated pri-miR-181a and pri-miR-181c to miR-181a and miR-181c. miR-181a and miR-181c can directly bind to the 3′ UTR of SFRP1 and promote osteogenic differentiation and inhibit adipogenic differentiation in BMSCs by negatively regulating the expression of SFRP1. The YTHDC1/miR-181a and miR-181c/SFRP1 axes may therefore be potential therapeutic targets for OP.

#### 3.1.12 miR-149-3p promotes osteogenic differentiation and inhibits adipogenic differentiation of BMSCs by targeting FTO (promoting bone formation and inhibiting adipogenesis)

Previous studies have also shown that miRNAs regulate BMSC differentiation (Xie et al., 2016). Li J. et al. (2019) used osteogenic induction media to culture BMSCs and found that the expression of miR-149-3p was significantly increased during the osteogenic differentiation of BMSCs. The upregulated expression of miR-149-3p participates in the osteogenic differentiation of BMSCs by promoting the expression of the osteogenic differentiation-related genes ALP, BGLAP, SPP1, COL1A1, and BMP4 in BMSCs. Li Y. et al. (2019) cultured BMSCs in adipogenic induction media and transfected them with miR-149-3p mimics to overexpress miR-149-3p. The results showed that the formation of lipid droplets in BMSCs was reduced and the expression of the adipogenic differentiation-related genes CEBPA, CEBPB, CEBPD, FABP4, and PPARG was significantly downregulated (Li J. et al., 2019). Thus, the adipogenic differentiation of BMSCs was inhibited. FTO was the target of miR-149-3p, and miR-149-3p regulated the osteogenic and adipogenic differentiation of BMSCs by the targeted inhibition of FTO expression. The expression of miR-149-3p in BMSCs can therefore positively regulate the expression of osteogenic differentiation-related genes and negatively regulate the expression of adipogenic differentiation-related genes by the targeted inhibition of FTO expression. This promotes the osteogenic differentiation and inhibits the adipogenic differentiation of BMSCs. Regulating the expression of miR-149-3p in BMSCs to restore the osteogenic-adipogenic balance of bone metabolism may be another direction for the clinical treatment of OP.

#### 3.1.13 TRAF4 promotes osteogenic differentiation and inhibits adipogenesis of MSCs by targeting ALKBH5 (contributing to bone formation and inhibiting adipogenesis)

Li Y. et al. (2019) found that during the osteogenic differentiation of MSCs, the expression levels of TRAF4, ALP activity, and calcium nodule formation were significantly enhanced, indicating that the expression of TRAF4 may be positively correlated with the osteogenic differentiation of MSCs. After using a lentivirus to transfect MSCs to overexpress TRAF4, the results showed that the formation of calcium nodules, ALP activity, and the expression of the osteogenesis-related genes RUNX2 and OCN in MSCs were significantly enhanced. The results following TRAF4 knockdown were the opposite. These results indicate that TRAF4 positively regulates the osteogenic differentiation of MSCs (Li J. et al., 2019). In addition, Cen et al. (2020) found that MSCs cultured in adipogenic media showed a decrease in the expression of TRAF4, while the protein levels of the adipogenic-related genes PPAR-γ, C/EBP-α, and FABP4 were significantly upregulated. This suggests that TRAF4 negatively regulates the adipogenic differentiation of MSCs (Cen et al., 2020). Further study showed that ALKBH5 positively regulated the expression level of TRAF4 by regulating the m6A methylation level of TRAF4 in MSCs, promoted the expression levels of the osteogenic-related genes RUNX2 and OCN in MSCs, and inhibited the expression levels of the adipogenic-related genes PPAR-γ, C/EBP-α, and FABP4 to regulate the osteogenic-adipogenic differentiation of MSCs (Cen et al., 2020). As an important influence node of MSC osteogenic and adipogenic differentiation, TRAF4 regulates the expression of TRAF4 by regulating the expression level of ALKBH5 and interferes with the osteogenic and adipogenic differentiation of MSCs, which may underscore a new way to prevent and treat patients with OP or bone loss in the future.

As a subset of MSCs, the regulatory mechanisms of m6A modifications in BMSCs may be as follows:

(1) METTL3 promotes OB-associated bone formation by the m6A methylation of VEGF in BMSCs or through the m6A-pre-miR-320/miR-320-RUNX2 axis; METTL14 induces autophagy and the OB differentiation of BMSCs *via* the IGF2BP/beclin-1 signalling axis; YTHDF1 regulates the OB differentiation of BMSCs by targeting VDAC3.
(2) FTO-mediated RUNX2 methylation levels negatively regulate the osteogenic differentiation of BMSCs.
(3) METTL3 promotes the OB differentiation of BMSCs and inhibits their adipoblastic differentiation through the PTH-PTH1R signalling axis; WTAP promotes the OB differentiation of BMSCs and inhibits their adipoblastic differentiation through the YTHDC1/miR-181a and miR-181c/SFRP1 signalling axes; miR-149-3p promotes the OB differentiation of BMSCs and inhibits their adipoblastic differentiation by targeting FTO.

### 3.2 Regulatory mechanisms of m6A on OBs

OBs originate from mesenchymal cells, accounting for 4%–6% of the total resident cells in the bone, and are the main functional cells in bone formation. Mature OBs are essential for matrix deposition and mineralisation (Bernar et al., 2022). OBs mainly undergo three transformation processes after their functions are completed: (1) undergoing apoptosis; (2) becoming endosteal cells; and (3) becoming osteocytes (Ponzetti and Rucci, 2021). OBs play an important role in the process of bone metabolism.

#### 3.2.1 METTL3 suppresses the osteogenic differentiation of MC3T3-E1 cells through the miR-7212-5p/FGFR3 signalling axis, inhibiting osteogenesis

Zhang et al. (Mi et al., 2020) constructed a femoral fracture mouse model by performing a transverse osteotomy in the middle of the femoral shaft in C57BL/6 J mice. Equal volumes of phosphate-buffered saline (PBS) and plasmid encoding METTL3 were locally injected into the fracture site on days 0, 4, and 7, and fracture healing was evaluated on days 14 and 21. The results showed that BV, TV, and BMD were significantly decreased in the plasmid METTL3 group, and the expression levels of osteogenesis-related genes (BMP2 and RUNX2) were also significantly decreased. Downregulating or silencing the expression of METTL3 could promote the osteogenesis process *in vitro* and *in vivo*, indicating that METTL3 overexpression inhibited the expression of osteogenesis-related genes in the femoral fracture mouse model, aggravated the damage to microstructure and morphology in the femoral fracture mouse model, and led to delayed fracture healing (Mi et al., 2020). The experimental results also showed that the change in miR-7212-5p expression during fracture healing in the femoral fracture mouse model was similar to that of METTL3. After measuring the pri-miRNA and miRNA levels in MC3T3-E1 cells and using a mouse OB precursor cell line transfected with plasmids METTL3 and si-METTL3, the expression level of miR-7212-5p in METTL3-overexpressing MC3T3-E1 cells was significantly enhanced, whereas the knockdown of METTL3 significantly reduced the expression level of miR-7212-5p in MC3T3-E1 cells (Mi et al., 2020). This confirmed that METTL3 could target and induce the expression of miR-7212-5p. After the transfection of MC3T3-E1 cells with agomir-7212-5p or antagomir-7212-5p, the level of miR-7212-5p significantly increased, while the osteogenic differentiation and matrix mineralisation of MC3T3-E1 cells were significantly inhibited (Mi et al., 2020). These results show that miR-7212-5p is a key factor regulating osteogenic differentiation and matrix mineralisation and is an important negative regulator of osteogenic differentiation. Further *in vivo* experiments revealed (Wang et al., 2022) that FGFR3 expression significantly increased after femoral fracture in mice. FGFR3 knockdown in MC3T3-E1 cells significantly inhibited the OB differentiation of MC3T3-E1 cells and reduced matrix mineralisation, indicating a positive regulatory role of FGFR3 in the process of osteogenic differentiation (Li et al., 2024). The results of this study indicate that FGFR3 may be a target of miR-7212-5p.

In conclusion, METTL3, a negative regulator of osteogenic differentiation, may regulate the osteogenic differentiation of OBs by targeting the METTL3/miR-7212-5p/FGFR3 signalling axis. Therefore, the knockdown of METTL3 may be a new measure to promote OB osteogenic differentiation and improve bone loss, which has guiding significance for the prevention and treatment of patients with OP and provides new insights into m6A modifications in OP.

#### 3.2.2 FTO promotes OB-associated bone formation through the HSPA1A/NF-κB signalling pathway

In addition to METTL3 playing a key regulatory role in OB differentiation, some demethylases also play important regulatory roles in OB osteogenic differentiation (Zhang et al., 2019; Huang et al., 2023). FTO was the first demethylase to be discovered. In addition to eliminating m6A modifications in RNA to regulate cell proliferation, differentiation, and apoptosis, it affects osteogenic differentiation by regulating adipogenesis (Huang et al., 2023). Zhang et al. (2019) constructed a mouse phenotype with an overall lack of FTO (Fto^KO^) or selective lack of FTO (Fto^OcKO^) in OBs. TUNEL assays were used to assess apoptosis of OBs in Fto^KO^ mice (Zhang et al., 2019). The data showed that the loss of FTO in OBs promoted apoptosis. The results further showed that FTO was necessary for the osteogenic differentiation of OBs. RNA profiling revealed that the overexpression of HSPA1A or inhibition of NF-κB signalling could normalise the DNA damage and apoptosis rates of OBs in Fto^KO^ mice, indicating that HSPA1A and NF-κB are downstream targets of FTO (Zhang et al., 2019). The overexpression of HSPA1A or inhibition of NF-κB signalling could protect OBs from apoptosis, thereby maintaining bone mass. The above results fully confirm that FTO inhibits OB apoptosis in Fto^KO^ mice by activating the expression of HSPA1A, a key player in the HSPA1A/NF-κB pathway, and inhibiting the expression of NF-κB, and ultimately participates in the regulation of bone metabolism in the body.

#### 3.2.3 ALKBH5 promotes OB-associated bone formation by enhancing the stability of RUNX2 mRNA

Feng et al. (2021) studied the function of ALKBH5 in the process of osteogenic differentiation and found that the mRNA and protein expression levels of ALKBH5 in OBs were significantly upregulated during OB differentiation. After ALKBH5 knockdown, its mRNA and protein levels in OBs, as well as ALP activity, extracellular matrix mineralisation, and expression of the osteogenic-related genes ALP, BSP, COL1A1, RUNX2, and Sp7, were significantly downregulated. These findings indicate that ALKBH5 has a positive effect on the osteogenic differentiation of OB cells (Feng et al., 2021). Primary OB cells were transfected with the NC plasmid, ALKBH5 plasmid, or ALKBH5 mutant plasmid (inactive in m6A demethylation) (Feng et al., 2021). The results showed that ALKBH5 was significantly upregulated in both the ALKBH5 plasmid group and the ALKBH5 mutant group. However, only ALP activity, extracellular matrix mineralisation, and the expression levels of the osteogenesis-related genes ALP, BSP, COL1A1, RUNX2, and Sp7 were significantly upregulated in the ALKBH5 plasmid group, which promoted OB differentiation (Feng et al., 2021). These results showed that ALKBH5 promoted OB differentiation depending on its demethylation activity. Following ALKBH5 knockdown (Feng et al., 2021), the expression of RUNX2 in OB cells decreased significantly, indicating a positive correlation between ALKBH5 and RUNX2 during osteogenic differentiation. RNA stability assay results showed that the half-life of RUNX2 mRNA in the ALKBH5 knockdown group was significantly shorter than that in the normal group, while the half-life of RUNX2 mRNA in the ALKBH5 overexpression group was significantly longer than that in the normal group; the half-life of RUNX2 mRNA in the mutant ALKBH5 was consistent with that in the NC group (Feng et al., 2021). These results indicate that ALKBH5 positively regulates the stability of RUNX2 mRNA through its demethylase activity, and the overexpression of ALKBH5 can enhance the stability of RUNX2 mRNA, thereby promoting osteogenic differentiation.

In summary, ALKBH5 promotes OB osteogenic differentiation by increasing the expression levels of the osteogenesis-related genes ALP, BSP, COL1A1, RUNX2, and Sp7. Moreover, ALKBH5 enhances the stability of RUNX2 mRNA through its demethylase activity, thereby promoting osteogenic differentiation. ALKBH5 may therefore be a key target for the regulation of osteogenic differentiation of OBs, as well as a key target for future research on the pathogenesis of OP or development of preventive drugs.

### 3.3 Regulatory mechanisms of m6A in osteoclasts

Osteoclasts are large, multinucleated cells derived from the hematopoietic lineage of monocytes and macrophages (Behzatoglu, 2021; Xian et al., 2012). They adhere to and degrade the bone matrix. During bone resorption, growth factors that accumulate in the bone matrix are released, triggering the recruitment of OBs responsible for new bone formation. Osteoclasts communicate closely with OBs under physiological conditions, participate in bone modelling and remodelling, and contribute to calcium homeostasis and bone immunity (Behzatoglu, 2021; Xian et al., 2012). They also play an important role in bone metabolism. Methyltransferases and demethylases participate in the regulation of bone metabolism by regulating osteoclast differentiation (Wang J. et al., 2023).

#### 3.3.1 METTL3 and ALKBH5 regulate the osteoclastic differentiation of osteoclasts *via* m6A methylation modifications targeting circ_0008542

Circ_0008542 is a circRNA present in the exosomes of MC3T3-E1 cells. Wang W. et al. (2021) stimulated exosomes with tension and found that the expression level of circ_0008542 continued to increase with the extension of tension time, indicating that the expression level of circ_0008542 was time-dependent upon tension stimulation. The transfection of circ_0008542 into RAW264.7, or the treatment with exosomes containing overexpressed circ_0008542 in RAW264.7 cells, resulted in a significant increase in the number of tartrate-resistant hydrochloric acid phosphatase (TRAP)-positive multinucleated cell bodies. Additionally, there was an increase in osteoclasts with enlarged F-actin bands, indicating that the area of resorption pits was significantly increased, and osteoclast activity was significantly enhanced (Wang J. et al., 2021). These results indicate that circ_0008542 positively regulates osteoclast differentiation and bone resorption. In the tension stimulation group (Wang W. et al., 2021), the expression levels of c-fos, NFATc1, RANK, and NF-κB p-p65 induced by RANKL were upregulated under the stimulation of exosomes, while the expression levels of CTSK, MMP9, and TRAP mRNA induced by RANKL were increased under the stimulation of exosomes (Wang J. et al., 2021). After the addition of exosomes under tension stimulation, the number and nuclei of TRAP-positive osteoclasts significantly increased, whereas the bone resorption area rate was downregulated (Wang W. et al., 2021). These results indicated that exosomes in the tension stimulation group positively regulated RANKL-induced osteoclast differentiation and function. In addition, the RNA pull-down test revealed that circ_0008542 was significantly enriched in the captured part of miRNA-185-5p, indicating that miRNA-185-5p was correlated with circ_0008542. Further, miRNA-185-5p mimics, miRNA-185-5p inhibitors, or siRNA were transfected into RAW264.7 cells (Wang J. et al., 2021). The results showed that osteoclast differentiation and bone resorption were inhibited when the expression of miRNA-185-5p was upregulated or when the expression of RANK was downregulated. It was further found that the biological response caused by the miRNA-185-5p inhibitor in RAW264.7 cells could be treated by the combination of the miRNA-185-5p inhibitor and siRANK. The osteoclast differentiation and bone resorption caused by the overexpression of circ_0008542 could be rescued by the combined use of miRNA-185-5p + circ_0008542 (Wang W. et al., 2021). These results showed that circ_0008542, a sponge for miRNA-185-5p, promoted osteoclast differentiation and bone resorption through the circ_0008542/miRNA-185-5p/RANK axis.

In a site-directed mutagenesis study, Wang J. et al. (2021) found that 1,956 bp in circ_0008542 was the m6A functional site. Methyltransferase METTL3 could change the spatial structure of the circ_0008542 gene through specific m6A functional sites and promote the binding of circ_0008542 and METTL3 to the target gene RANK. METTL3, dependent on its mediated m6A methylation, could serve as a node to control the “m6A switch” of circ_0008542 1956bp, promoting the binding efficiency between circ_0008542 and miRNA-185-5p and promoting RANK expression to positively regulate osteoclast differentiation and bone metabolism. The study also (Wang J. et al., 2021) transfected circ_0008542 or circ_0008542+si-METTL3/ALKBH5 into RAW264.7 cells and circ_0008542-overexpressing exosomes were added to RAW264.7 cells. The results showed that osteoclast differentiation and bone resorption significantly increased in the circ_0008542 group, whereas osteoclast differentiation and bone resorption significantly decreased in the circ_0008542 + si-METTL3/ALKBH5 group. RANK-overexpressing cells and exosomes (MC3T3-E1 cells treated with circ_0008542 si-METTL3/ALKBH5) were transfected into RAW264.7 cells (Wang W. et al., 2021). The experimental results showed that, compared with the circ_0008542 + si-METTL3/ALKBH5 group, osteoclast differentiation and bone resorption in the circ_0008542 + si-METTL3/ALKBH5 + RANK group were significantly increased (Wang J. et al., 2021). The above experimental results showed that the upregulation of METTL3 or downregulation of ALKBH5 could positively regulate the m6A methylation level of circ_0008542, promote the binding efficiency between circ_0008542 and miRNA-185-5p, and promote RANK expression to positively regulate osteoclast differentiation and bone metabolism (Wang J. et al., 2021). ALKBH5 acts as an “m6A switch” for circ_0008542 1956bp *via* past methylation, inhibiting the binding of circ_0008542 to the miRNA-185-5p/RANK axis. Therefore, inhibiting the binding of circ_0008542 to miRNA-185-5p by reducing the molecular sponge effect of circ_0008542 may correct excessive bone resorption (Huang et al., 2021). Inhibiting METTL3 or overexpressing ALKBH5, the effect of the “m6A switch,” and inhibiting osteoclast differentiation and bone resorption may be other directions for the clinical inhibition of bone loss.

**FIGURE 3:**
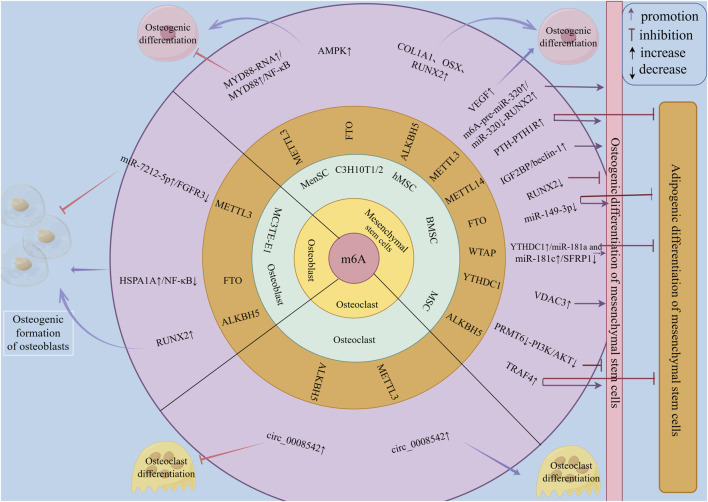
The regulatory role of m6A family members in bone metabolism of osteoporosis. m6A family members regulate bone metabolism in osteoporosis at the RNA level through m6A methylation, mainly by modulating the osteogenic and adipogenic differentiation of mesenchymal stem cells (MSCs) to affect bone metabolism in osteoporosis. They can also influence bone metabolism in osteoporosis by regulating the osteogenic differentiation and formation of osteoblasts, as well as the osteoclastic differentiation and resorption of osteoclasts.

In summary, m6A family proteins can influence the bone metabolism process in OP by regulating multiple targets and regulatory pathways of MSCs in osteogenic and adipogenic differentiation, OBs in osteogenic differentiation and bone formation, and osteoclasts in osteoclastic differentiation and bone resorption (Figure 3). Therefore, they may serve as a new direction for guiding the clinical prevention and treatment of osteoporosis.

## 4 Targeted therapy

At present, several achievements have been made in the field of m6A methylation-based treatments and the prevention of OP, with small-molecule targeted drugs and exosomes being important research topics.

### 4.1 METTL3 inhibitors

**FIGURE 4:**
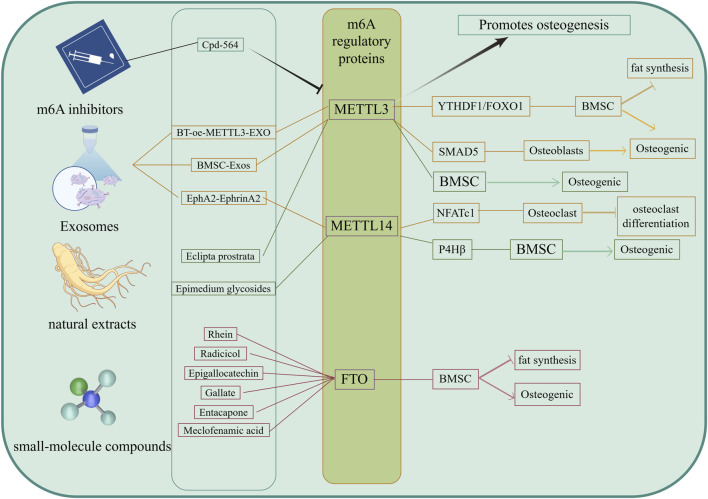
Targeted therapy of m6A. The Drugs of targeting m6A were included METTL3 inhibitors, exosomes, small molecule compounds and naturalextracts. These targeted drugs mainly affect the m6A modification level of mRNA by acting on m6A regulatory proteins, further promote osteogenic differentiation of BMSCs and inhibit adipogenic differentiation, as well as promote osteogenic differentiation and inhibit osteoclast differentiation, ultimately delaying bone loss.

Cpd-564 is one of the METTL3 inhibitors, and it has been found (Liu et al., 2024) that Cpd-564 can significantly reduce the mRNA expression of osteoblast senescence proteins p16 and p21, promote the expression of osteoblast cyclin Ccnd1, and inhibit osteoblast senescence. When Cpd-564 was injected into 24-month-old rats, it was found that the expression of METTL3 in Cpd-564-treated 24-month-old rats was significantly downregulated compared with that in 2-month-old rats, and the volume, thickness, and number of trabecular bone in femur tissue of 24-month-old rats treated with Cpd-564 were significantly increased (Liu et al., 2024). *In vivo* and *in vitro* experiments showed that Cpd-564 could inhibit the expression of METTL3, thereby inhibiting osteoblast senescence and delaying the occurrence and progression of osteoporosis, which is a promising new method for the prevention of osteoporosis (Figure 4).

### 4.2 Exosomes

Exosomes are nanoscale bilayer lipid vesicles (50–150 nm in diameter) that can deliver a variety of small molecules and trigger minimal immune rejection, playing an increasingly important role in the treatment of OP (Sun et al., 2019). Yang et al. (2023) have reported that METTL14 released from exosomes enhances the m6A methylation level of NFATc1, thereby inhibiting osteoclasts. Both *in vitro* and *in vivo*, osteoclasts could take up more METTL14 from exosomes through the overexpression of the EphA2 receptor on the surfaces of OB exosomes, while there were corresponding EphrinA2 ligands on the surfaces of osteoclasts. OBs and osteoclasts transmitted biological signals through the EphA2-EphrinA2 pathway (Yang et al., 2023). The overexpression of EphA2 in exosomes may therefore be an effective biological carrier for the targeted inhibition of osteoclasts, which can help patients with OP maintain bone mass while avoiding the side effects of bisphosphonates. This also suggests an effective method for targeting and inhibiting osteoclast bone resorption to maintain bone mass and avoid bone loss, which has guiding significance for the clinical treatment and prevention of OP. By targeting the inhibition of osteoclast bone resorption, Li T. et al. (2025) and colleagues developed serum-derived exosomes that were named BT-oe-METTL3-EXO. These exosomes were modified by loading abundant METTL3 mRNA and decorating their surfaces with targeted bone peptides. They not only contained high levels of METTL3 but also exhibited excellent bone-targeting efficiency. METTL3 worked in tandem with YTHDF1 in the serum-derived exosomes, enhancing FOXO1 gene transcription by promoting the m6A modification of FOXO1, which promotes OB differentiation in BMSCs while inhibiting their adipogenic differentiation. *In vitro* studies have shown that BT-oe-METTL3-EXO can significantly reduce bone loss induced by ovarianectomy in mice, enhance OB differentiation of BMSCs, and inhibit adipogenic differentiation. Li Z. et al. (2025) and colleagues also found that exosomes METTL3 derived from bone marrow mesenchymal stem cells (BMSC-derived exosomes, BMSC-Exos) regulated SMAD5 m6A methylation, promoting OB differentiation. Exosomes can target BMSCs, OBs, or osteoclasts as needed, regulating the expression of m6A regulatory proteins within cells to promote OB differentiation and inhibit osteoclastic resorption, thereby effectively maintaining bone mass and preventing bone loss. These findings have significant implications for the clinical management and prevention of OP (Figure 4).

### 4.3 Others (small-molecule compounds, natural extracts, Chinese patent medicines)

FTO is an m6A demethylase. Previous studies have shown that (Wang W. et al., 2021) the overexpression of FTO inhibits the OB differentiation of BMSCs and promotes their adipogenic differentiation, suggesting that FTO may be a novel therapeutic target for OP. Its inhibitors may be used to prevent and treat OP. Currently, the main small-molecu。 inhibitors of FTO include Rhein, Radicicol, Epigallocatechin, Gallate, Entacapone, and Meclofenamic acid, among others (Li J. et al., 2022). Recent studies have also found that some natural extracts can participate in bone metabolism. For example, Epimedium glycosides can upregulate the m6A modification of the P4Hβ subunit of prolyl 4-hydroxylase mediated by METTL14, thereby promoting the OB differentiation of BMSCs (Jin et al., 2024). The extract and component wedelolactone from *Eclipta prostrata* (L.) L. enhances the OB generation of BMSCs by targeting the RNA m6A methylation mediated by METTL3 (Tian et al., 2023) (Figure 4).

## 5 Summary and outlook

**FIGURE 5:**
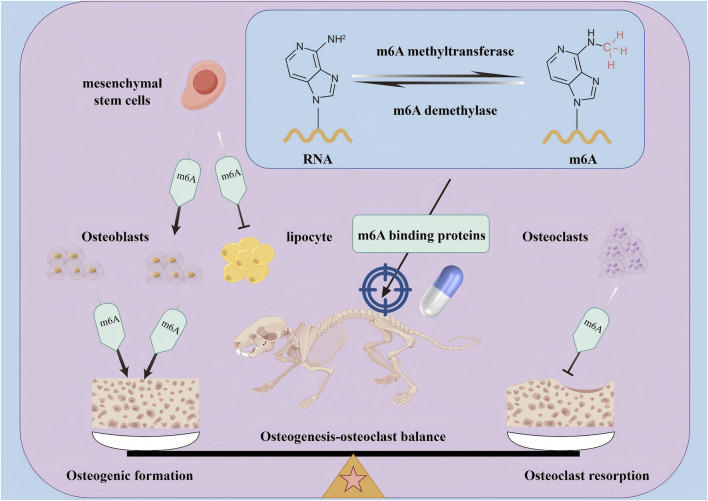
The mechanism of m6A and m6A targeted therapy in regulating osteogenesis- osteoclast balance. The m6A regulatory factors (including m6A methyltransferase, m6A demethylase, and m6A binding protein) mainly regulate the transcription and translation processes of mRNA by adjusting the levels of m6A on mRNA, thereby targeting the dynamic balance between osteoblasts and osteoclasts in OP bone metabolism, and ultimately exerting a therapeutic role in treating OP.

Osteoporosis is a chronic metabolic bone disease with an unknown mechanism and a lack of effective treatment. This review found that m6A methyltransferase, m6A demethylase, and m6A-binding protein regulate osteogenic and adipogenic differentiation of mesenchymal stem cells, osteogenic differentiation of osteoblasts, and osteoclast differentiation of osteoclasts by affecting m6A levels of mRNA. In addition, this review also summarises that m6A-targeted drugs can promote the osteogenic differentiation of mesenchymal stem cells, inhibit adipogenic differentiation, promote osteogenic differentiation of osteoblasts, and inhibit osteoclast differentiation by regulating the expression of m6A methyltransferase, m6A demethylase, or m6A-binding protein, and ultimately restore osteogenesis-osteoclast homeostasis and delay the occurrence and progression of osteoporosis (Figure 5). At present, this review has also found that some m6A-based targeted therapies, such as METTL3 inhibitors, exosomes, small molecule compounds, and biological extracts, have achieved satisfactory results in restoring osteogenesis-osteoclast homeostasis. This therapy is expected to improve the quality of life of patients, provide multi-target intervention, reduce the side effects of Western medicine treatment, effectively solve the complex pathology of OP, and improve the treatment effect.

Several challenges and limitations persist. As reported in a previous study, the total level of m6A in the callus of C57BL/6 J mouse femoral fracture models did not significantly decrease within the first 7 days of fracture and then gradually increased as the fracture healed (Mi et al., 2020), indicating that m6A modifications may promote bone formation as a whole. The specific underlying mechanisms, however, remain inconclusive. m6A may have different or dual effects on the body under different microenvironments, acting on different targets and being affected by different regulatory factors. The interactions between m6A methylation and bone metabolism are complex. The restoration of bone metabolic homeostasis can regulate aberrant m6A methylation. In contrast, abnormal levels of m6A methylation can regulate signalling pathways, transcription factors, and metabolic enzymes related to bone metabolic balance. In tumour studies, METTL3 has been found to act as an oncogene in acute myeloid leukaemia but as a tumour suppressor in glioblastoma multiforme. The inhibition of specific m6A regulators may induce the development of other types of cancer or other diseases (Sun et al., 2019). Some proteins related to m6A also exhibit similar functions in bone metabolism. Different genes or proteins may have opposing effects in different environments. m6A plays a complex dual role in promoting or inhibiting OB formation and osteoclast resorption during bone metabolism.

The key role of m6A mRNA methylation in the occurrence and development of OP has provided new possibilities for its early diagnosis and treatment. However, the specific roles and mechanisms of m6A methylation in bone metabolism have not yet been elucidated. Therefore, more in-depth research on the mechanisms of m6A methylation is required.

There are many challenges in research on m6A mRNA methylation. m6A-related proteins may affect the bone metabolic processes in an m6A-independent manner. Li et al. (2023) found that hsa_circ_011458 promotes the expression of HNRNPA3 in BMSCs by targeting the upregulated expression of miR-155-5p, ultimately promoting the osteogenic differentiation of BMSCs. HNRNPA3 is an m6A-binding protein, but its role has not yet been elucidated in relation to m6A methylation. Additionally, little is known about the mechanisms that control the expression and activity of m6A methyltransferases, demethylases, and m6A-binding proteins in different tissues, cells, and even microenvironments. For example:

(1) In BMSCs, the expression of METTL3 promotes OB differentiation (Zhou et al., 2024). However, in MC3T3-E1 cells, METTL3 not only inhibits OB senescence (Wang et al., 2024) but also suppresses the expression of osteogenesis-related genes (Mi et al., 2020). This effect may be due to multiple factors working together. First, BMSCs and MC3T3-E1 cells belong to different cell lines. BMSCs are MSCs with the potential to differentiate into various cell types, including OBs, adipocytes, and chondrocytes. MC3T3-E1 cells are mouse embryonic OBs that primarily differentiate into mature OBs and participate in bone matrix formation and mineralisation. The distinct characteristics and functions of these two cell types result in different mechanisms and backgrounds for the role of METTL3, leading to varied outcomes. Secondly, the overexpression of METTL3 in BMSCs can promote the m6A modification of Bcl-2, Mcl-1, and BIRC5 mRNA, enhance the binding of IGF2BP2 to these mRNAs, increase their stability, and inhibit hypoxia-induced apoptosis in BMSCs (Xie et al., 2024). Additionally, METTL3 can promote OB differentiation in BMSCs through the IGF2BP1/m6A/RUNX2 signalling pathway (Zhou et al., 2024). In MC3T3-E1 cells, METTL3 enhances the stability of Hspa1a mRNA through m6A modifications, thereby inhibiting OB senescence (Wang et al., 2024). METTL3 can also inhibit the activation of the Wnt/β-catenin signalling pathway in MC3T3-E1 cells, thus preventing oxidative stress-induced OB apoptosis (Yang et al., 2025). Furthermore, high sugar and fat levels may induce iron death in OBs *via* the METTL3/ASK1-p38 signalling pathway (Lin et al., 2022). Knocking down METTL3 in MC3T3-E1 cells inhibits OB differentiation and mineralisation. These findings demonstrate that METTL3 regulates OB differentiation differently in various cells and even within the same cell under different conditions, suggesting a mechanism closely linked to complex signalling networks.
(2) The occurrence of OP is closely related to patient age. A key feature of OP caused by age-related bone loss is the accumulation of senescent OBs in the bone microenvironment. METTL3 deficiency can inhibit OB senescence and the accumulation of senescent OBs. Patient age and OB senescence are important factors when considering the roles and mechanisms of m6A methylation and related proteins.
(3) The role of FTO in OP remains controversial. However, FTO knockdown or overexpression in BMSCs can reduce osteogenic differentiation. While FTO in OVX mice can promote bone formation and alleviate OP, BMD and the bone mineral content in normal bones are difficult to maintain after FTO knockout (Huang et al., 2023). These results suggest that FTO may play a dual role in bone metabolism and the maintenance of bone homeostasis to a certain extent; however, the key points of this process are not completely clear. In addition, animal studies have indicated that FTO may be a potential biomarker of OP. However, it is unclear if FTO can play the same role in patients with OP, making further validation needed *via* clinical studies.
(4) TRAF4 acts as an E3 ubiquitin ligase and positively regulates the osteogenic differentiation of MSCs by ubiquitinating the K119 site of Smurf2 through the K48 ubiquitin chain and degrading Smurf2 (Li Y. et al., 2019). It also activates β-catenin signal transduction to negatively regulate the adipogenesis of MSCs by interacting with PKM2 (Cen et al., 2020). As an important influence node of MSC osteogenic and adipogenic differentiation, TRAF4 regulates the expression of TRAF4 by regulating the expression level of ALKBH5. Further research is needed to determine whether ALKBH5 specifically targets and regulates TRAF4 expression or whether a relationship exists between TRAF4 mRNA and m6A methylation that utilises the demethylase activity of ALKBH5 to exert regulatory effects.

Finally, most current studies on the role and mechanisms of m6A methylation are limited to single tissues, single pathways, or even single cells. Studying the complex effects and mechanisms of m6A methylation on different proteins, cells, tissues, and microenvironments in the body is an exciting field of research and holds promise in the treatment of OP.
